# *CrMAPK3* regulates the expression of iron-deficiency-responsive genes in *Chlamydomonas reinhardtii*

**DOI:** 10.1186/s12858-017-0081-5

**Published:** 2017-05-16

**Authors:** Xiaowen Fei, Junmei Yu, Yajun Li, Xiaodong Deng

**Affiliations:** 1 School of Science, Hainan Medical College, Haikou, 571101 China; 2 Institute of Tropical Bioscience and Biotechnology, Chinese Academy of Tropical Agricultural Science, Key Laboratory of Tropical Crop Biotechnology, Ministry of Agriculture, Haikou, 571101 China

**Keywords:** *Chlamydomonas reinhardtii*, Mitogen-activated protein kinases, Iron deficiency, Real-time PCR

## Abstract

**Background:**

Under iron-deficient conditions, *Chlamydomonas* exhibits high affinity for iron absorption. Nevertheless, the response, transmission, and regulation of downstream gene expression in algae cells have not to be investigated. Considering that the MAPK pathway is essential for abiotic stress responses, we determined whether this pathway is involved in iron deficiency signal transduction in *Chlamydomonas*.

**Results:**

*Arabidopsis* MAPK gene sequences were used as entry data to search for homologous genes in *Chlamydomonas reinhardtii* genome database to investigate the functions of mitogen-activated protein kinase (MAPK) gene family in *C. reinhardtii* under iron-free conditions. Results revealed 16 *C. reinhardtii* MAPK genes labeled *CrMAPK2*–*CrMAPK17* with TXY conserved domains and low homology to MAPK in yeast, *Arabidopsis*, and humans. The expression levels of these genes were then analyzed through qRT-PCR and exposure to high salt (150 mM NaCl), low nitrogen, or iron-free conditions. The expression levels of these genes were also subjected to adverse stress conditions. The mRNA levels of *CrMAPK2*, *CrMAPK3*, *CrMAPK4*, *CrMAPK5*, *CrMAPK6*, *CrMAPK8*, *CrMAPK9*, and *CrMAPK11* were remarkably upregulated under iron-deficient stress. The increase in *CrMAPK3* expression was 43-fold greater than that in the control. An RNA interference vector was constructed and transformed into *C. reinhardtii* 2A38, an algal strain with an exogenous *FOX1*:*ARS* chimeric gene, to silence *CrMAPK3*. After this gene was silenced, the mRNA levels and ARS activities of *FOX1*:*ARS* chimeric gene and endogenous *CrFOX1* were decreased. The mRNA levels of iron-responsive genes, such as *CrNRAMP2*, *CrATX1*, *CrFTR1*, and *CrFEA1*, were also remarkably reduced.

**Conclusion:**

*CrMAPK3* regulates the expression of iron-deficiency-responsive genes in *C. reinhardtii*.

## Background

*Chlamydomonas reinhardtii* (Volvocales, Chlorophyta) is a single-celled eukaryotic and flagellated green alga, whose three genetic systems located in the nucleus, chloroplast, and mitochondria can be used for transformation. This alga is regarded as a “photosynthetic yeast” because of its easy culturing process, rapid growth, short life cycle, and high photosynthetic efficiency. With its three genome sequences, this model organism is highly useful for cell and molecular biology research [1].

In phosphorylation cascades, mitogen-activated protein kinases (MAPKs) are eukaryotic signal proteins involved in extracellular signal amplification and intracellular signal transduction in yeasts, animals, and plants [2–4]. Combined with other signal molecules, MAPKs transfer external stimuli via successive phosphorylation reactions: MAPKKKs → MAPKKs → MAPKs. Progressively and continuously enlarged signals, such as environmental stress factors, including high salinity, high osmotic pressure, and low temperature, reach the nucleus and regulate downstream gene expression [5, 6]. In eukaryotic cells, phosphorylation cascades are composed of MAPKs, MAPKKs, and MAPKKKs. *Homo sapiens* possesses 15 MAPKs, 7 MAPKKs, and 16 MAPKKKs, while *Arabidopsis* contains 20 MAPKs, 10 MAPKKs, and 80 MAPKKKs. Few MAPK cascades have been described because of the complexity of genetic networks and pleiotropic and interaction effects. MAPK genes have been identified in plants, such as *Arabidopsis*, rice, corn, wheat, and barley [7–13]. MAPKs function through stress-response pathways [14, 15].

Iron is an essential trace element for most living organisms. A precise iron regulation system is necessary to maintain the dynamic equilibrium of iron [16] because iron overload and deficiency cause metabolic disorders. Following nitrogen and phosphate deficiencies, iron deficiency restricts plant growth and yield and consequently induces crop chlorosis and yields low productivity. In humans, insufficient iron concentrations trigger iron deficiency anemia or iron deficiency syndrome. Iron has also been considered a growth-limiting factor in some tumor cells. Therefore, iron chelators are clinically used for cancer suppression.

Under iron-deficient conditions, *Chlamydomonas* exhibits high affinity for iron absorption that slightly differs from iron absorption in plants. Environmental ferric iron is reduced to ferrous iron via FRE1 (homology of *Arabidopsis* FRO2 [17]) on the plasma membrane and then putatively transferred to FOX1 by FEA1 [18]. Afterward, FOX1 oxidizes ferrous iron to ferric iron, which is then transported to the cytoplasm by FTR1 on the plasma membrane [19–21]. The expression of the genes encoding these proteins is significantly increased under iron-deficient conditions, and this phenomenon indicates that iron deficiency signals in these genes are regulated. Nevertheless, the response, transmission, and regulation of downstream gene expression in algal cells have yet to be investigated. Considering that the MAPK pathway is essential for non-biological stress responses, we determined whether this pathway is involved in iron deficiency signal transduction in *Chlamydomonas*. In this study, *Arabidopsis* MAPKs were used to search for the corresponding genes in the *Chlamydomonas* genome database (https://phytozome.jgi.doe.gov/pz/portal.html #), and 16 homologous genes, namely, CrMAPK2–CrMAPK17, were obtained. The mRNA expression level variation of these genes exposed to different stressors, such as –Fe, −N, and osmotic shock (150 mM NaCl), was also detected. Among these genes, *CrMAPK3* is specifically functionally analyzed by RNA silencing.

## Results

### Bioinformatics Analysis of MAPK Genes in Chlamydomonas

**Fig. 1:**
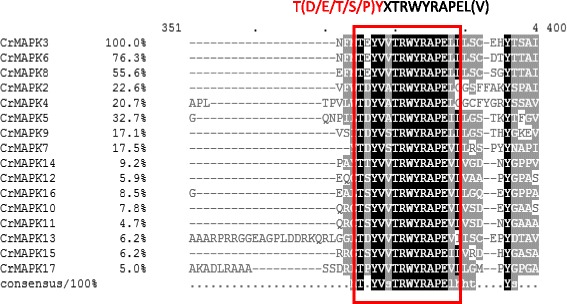
Multiple alignments of T(D/E/T/S/P)Y domains from MAPK proteins. The U-box domains in CrPUB proteins were predicted using MEME programs. Their sequences were aligned using ClustalX 2.1, and the alignments were edited using GeneDoc 2.7 sequence editor. Black, gray, and light gray shades indicate the identities and similarities among these sequences as 100, 80, and 60%, respectively

**Fig. 2:**
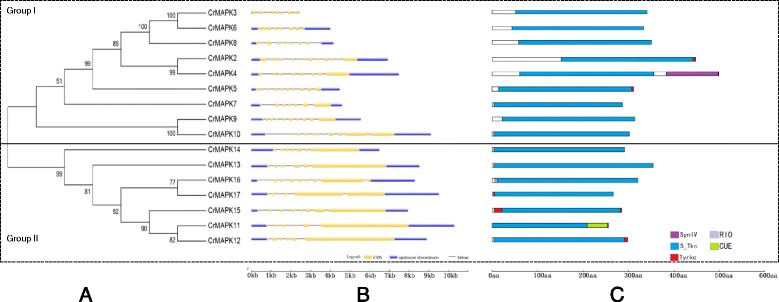
An analytical view of the CrMAPK gene family. **a** An unrooted tree summarizing the evolutionary relationships among the 16 members of the CrMAPK family. Multiple alignments of the 16 CrMAPK protein sequences from C. reinhardtii were conducted using ClustalX 2.1. The phylogenetic tree was constructed using MEGA6. The numbers on each node are Shimodaira-Hasegawa-like test indices of statistical support provided by MEGA6. Bar = 2.0 is a branch length that represents the number of amino acid substitutions per site. The tree shows the 3 phylogenetic subfamilies (numbered I to III and marked with different color backgrounds) with high predictive values. **b** Intron/exon structures: the gene structures were drawn using the online tool GSDS. As shown in the legend, the exons and introns are indicated by green rectangles and thin lines, respectively. The untranslated regions (UTRs) are indicated by blue boxes. The sizes of exons and introns can be estimated using the scale shown at the bottom. **c** Schematic representation of the conserved motifs in the 16 CrMAPK proteins elucidated using SMART and PROSITE online. The different domains are indicated by different colored boxes denoted at the bottom right corner. The lengths of the proteins and motifs can be estimated using the scale shown at the bottom

Sixteen homologous genes (Table 1), which are localized in chromosomes 1, 2, 3, 8, 12, 13, 16, and 17, were identified by searching the *Chlamydomonas* genome database with Blast. The predicted open reading frames of these genes were 1062–5301 bp in length, and their protein products contained 353–1766 amino acids with molecular weights of 39.8–178.76 kD and isoelectric points of 5.68–9.5. Fourteen of the MAPKs located in the cytosome were predicted by Euk-mPLoc2.0 except *CrMAPK6* and *CrMAPK14*, which exist in the nucleus. Using PROSITE predictions, we verified that the 16 *CrMAPKs* were mitogen-activated protein kinases. Multi-sequence alignment of the MAPK-specific TXY motifs in the CrMAPK proteins revealed that the T(D/E/T/S/P)Y activation loop motifs were conserved in the serine-threonine kinase (S-Tkc) domain in the 16 *CrMAPKs* (Fig. 1). *CrMAPK3*, *CrMAPK6*, and *CrMAPK8* contain TEY; *CrMAPK2*, *CrMAPK4*, *CrMAPK5*, *CrMAPK7*, *CrMAPK9*, *CrMAPK10*, and *CrMAPK13* comprise TDY; *CrMAPK11*, *CrMAPK12*, *CrMAPK15*, and *CrMAPK16* possess TSY; *CrMAPK17* is composed of TPY; and *CrMAPK14* consists of TTY. *Chlamydomonas* MAPKs were divided into two groups by using MEGA6. Group I contained *CrMAPK2* to *CrMAPK10*, whereas Group II comprised *CrMAPK11* to *CrMAPK17* (Fig. 2a). All of the MAPK genes contained 6 to 10 exons. The gene length ranged from 2.8 kb to 10.5 kb. Among these genes, *CrMAPK3* is the shortest and *CrMAPK11* is the longest (Fig. 2b). In addition to the S-Tkc-conserved region, 8 other protein domains/motifs, such as Syn N(Syntaxin N-terminal domain), RIO (RIO-like kinase), CUE (domain that may be involved in binding ubiquitin-conjugating enzymes), and Tyr-kc (tyrosine kinase catalytic domain) motifs (Fig. 2c), are present in *CrMAPKs*. The annotated *CrMAPK1*(Cre13.g582650) in JGI database is a small protein with 149 aa. After the alignment was compared with the other *CrMAPKs*(CrMAPK2-*CrMAPK17*), the results revealed that the conserved domain (T(D/E/T/S/P)YXTRWYRAPEL(V)) in the MAPK family could not be found in *CrMAPK1*. As such, *CrMAPK1* is not included in Table 1.

**Table 1:** List of the 16 MAPK genes identified in C. reinhardtii and their sequence characteristics

Name	Locus Name	ORF (bp)	Amino Acids	kD	pI	Chromosomal localization	Sub cellular location
CrMAPK2	Cre08.g385050	2223	740	79.0	8.61	chr8:4906426..4913322 F	Cytoplasm
CrMAPK3	Cre12.g509000	1062	353	39.8	8.76	chr12:2119590..2122035 R	Cytoplasm
CrMAPK4	Cre17.g745447	2298	765	81.2	7.20	chr17:6855222..6862749 F	Cytoplasm
CrMAPK5	Cre13.g607300	1302	433	48.5	8.88	chr13:5094976..5099387 F	Cytoplasm
CrMAPK6	Cre12.g508900	1128	375	42.5	7.66	chr12:2129460..2133325 R	Nuclear
CrMAPK7	Cre16.g661100	1758	585	63.3	9.7	chr16:2519399..2524020 F	Cytoplasm
CrMAPK8	Cre01.g010000	1170	389	44.0	5.68	chr1:1838122..1842287 F	Cytoplasm
CrMAPK9	Cre12.g538300	1923	640	68.4	9.1	chr12:6473633..6479228 F	Cytoplasm
CrMAPK10	Cre01.g052800	3692	1163	117	9.91	chr1:7342585..7351700 R	Cytoplasm
CrMAPK11	Cre01.g052850	5199	1732	168	9.5	chr1:7356808..7367075 F	Cytoplasm
CrMAPK12	Cre03.g200200	5301	1766	178	9.36	chr3:8184080..8192783 R	Cytoplasm
CrMAPK13	Cre10.g432250	4752	1583	160	8.31	chr10:1948371..1956866 R	Cytoplasm
CrMAPK14	Cre03.g169500	3321	1106	114	9.49	chr3:3709775..3716106 R	Nuclear
CrMAPK15	Cre02.g111014	4212	1403	142	9.15	chr2:5820048..5827789 F	Cytoplasm
CrMAPK16	Cre17.g709500	3237	1078	107	7.93	chr17:1790415..1798683 R	Cytoplasm
CrMAPK17	Cre17.g709750	4956	1651	165	8.56	chr17:1821974..1831590 F	Cytoplasm

F and R represent the forward and reverse directions on the chromosome, respectively. In total, 16 CrMAPK proteins were obtained by BLASTP search using the C. reinhardtii V5.5 proteome database and MAPK proteins from Arabidopsis thaliana as queries. The 16 CrMAPK genes were named based on their name annotated in JGI database. The molecular weights and pIs of the 16 CrMAPK proteins were predicted using ExPASy. The CrMAPK sub-cellular locations were predicted using the Euk-mPLoc2.0 program

### Analysis of mRNA levels of MAPK gene under − Fe, −N, and 150 mM NaCl stress conditions

**Fig. 3:**
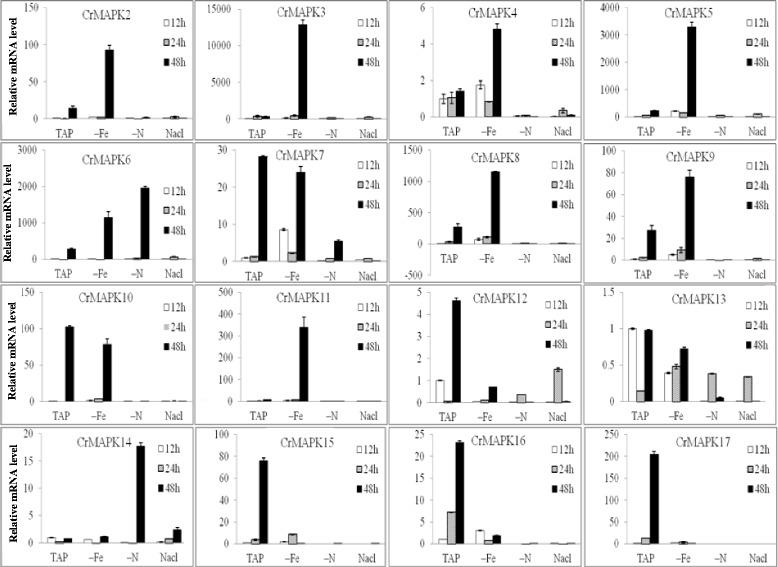
Results of qPCR analysis of the CrMAPK genes under − Fe, −N, or 150 mM NaCl conditions. C. reinhardtii CC425 was pre-cultured in TAP to the mid-logarithmic phase, followed by centrifugation and resuspension in TAP, TAP-Fe, TAP-N, and TAP with 150 mM NaCl. All the samples continued culturing for 12, 36, and 48 h. The cells were collected, and RNA samples were isolated. The gene transcript levels were determined using Real Time quantitative PCR. All expression values were normalized to the value of the 18S rRNA gene. The relative amounts were calibrated based on the number of transcripts of the corresponding genes in cells maintained in TAP for 12 h

The RNA extracted from the samples of *Chlamydomonas* cultivated under − Fe, −N, and 150 mM NaCl conditions was used for quantitative analysis, and the results are shown in Fig. 3. *CrMAPK2*–*CrMAPK17* expression levels were affected by iron deficiency, nitrogen deficiency, and high salt concentration. Compared with the expression of the gene in the TAP medium, the mRNA expression levels of *CrMAPK2*, *CrMAPK3*, *CrMAPK4*, *CrMAPK5*, *CrMAPK6*, *CrMAPK8*, *CrMAPK9*, and *CrMAPK11* were increased by iron deficiency to various degrees. *CrMAPK3*, *CrMAPK5*, and *CrMAPK11* respectively increased by 43-, 5-, and 40-fold after cultivation for 48 h. However, iron deficiency decreased the mRNA levels of *CrMAPK7*, *CrMAPK12*, *CrMAPK13*, *CrMAPK15*, *CrMAPK16*, and *CrMAPK17* after cultivation for 48 h. In nitrogen deficiency, the mRNA expression levels of *CrMAPK6* and *CrMAPK14* were significantly increased, whereas the expression levels of most MAPKs, such as those of *CrMAPK2*, *CrMAPK3*, *CrMAPK5*, *CrMAPK5*, *CrMAPK8*, *CrMAPK9*, *CrMAPK10*, *CrMAPK11*, *CrMAPK12*, *CrMAPK13*, *CrMAPK15*, *CrMAPK16*, and *CrMAPK17*, of *C. reinhardtii* were inhibited, and the mRNA expression levels of these genes were significantly decreased. The mRNA expression levels of all MAPK genes were also inhibited under high salt (150 mM NaCl) condition, and the mRNA expression of *CrMAPK17* was reduced by 10E10.

### CrMAPK3 positively regulates the expression of CrFOX1 gene

**Fig. 4:**
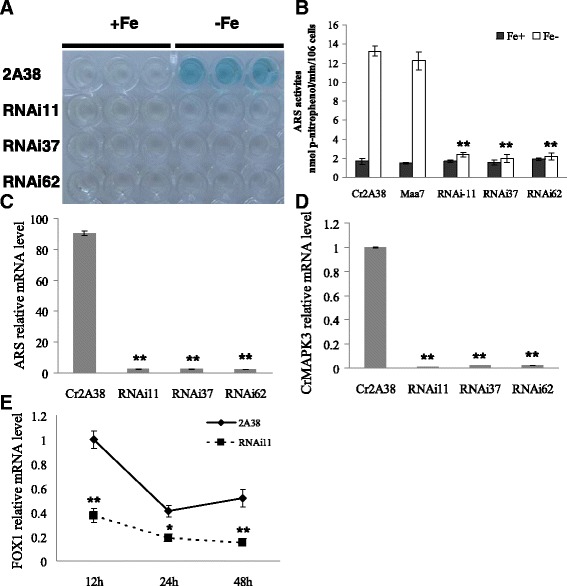
Analysis of CrMAPK3 RNAi transgenic algal strains. Of the 133 CrMAPK3 RNAi transformants, 99 were colorless or light blue. Among them, the ARS activities of transgenic strains RNAi11, RNAi37, and RNAi62 were significantly decreased under − Fe (**a**, **b**). Moreover, the mRNA levels of ARS were significantly decreased (**c**). The mRNA level of target gene CrMAPK3 was decreased by 97–98% compared with the control (**d**), indicating that CrMAPK3 in transgenic strains of RNAi11, RNAi37, and RNAi62 has been effectively silenced. The mRNA levels of endogenous CrFOX1 were reduced by 63, 54, and 71%, respectively, at 12, 24, and 48 h post-incubation in –Fe, indicating that CrMAPK3 positively regulates the expression of CrFOX1 gene (**e**). The data are shown as the means (±SD, *n* = 3). Significance is indicated as *P < 0.05, **P < 0.01

The *C. reinhardtii* 2A38 strain was prepared by using the integrated *FOX1* promoter:*ARS* box into the chromosome of the CC425 strain. Under iron-deficient conditions, *CrFOX1* promoted the ARS reporter expression and appeared deep blue when this gene was mixed with XSO4 substrate or yellow when this gene was mixed with *p*-nitrophenylsulfate. A total of 133 colonies were obtained after *Maa7IR*/*CrMAPK3IR* was transformed into *C. reinhardtii* 2A38 and then transferred onto –Fe plates with XSO4 to determine the ARS activities. Only 44 colonies and the control sample of 2A38 appeared blue, whereas the 99 other colonies were colorless or light blue. Furthermore, 74.4% of chromogenic reactions indicated that *CrMAPK3* silencing affected the FOX1 promoter function in –Fe. The transformants of RNAi11, RNAi37, and RNAi62 appeared colorless in both + Fe (16 uM) and –Fe except the non-transgenic *C. reinhardtii* 2A38 control, which appeared deep blue under –Fe conditions (Fig. 4a). These results were further confirmed by the ARS activity in transgenic strains. The ARS activities of RNAi11, RNAi37, and RNAi62 respectively decreased by 82, 85, and 83% compared with those of the *C. reinhardtii* 2A38 control (Fig. 4b). The mRNA of the ARS2 of the transgenic stains decreased by more than 97% in –Fe (Fig. 4c). The mRNA levels of the target *CrMAPK3* of the transgenic strains RNAi11, RNAi37, and RNAi62 decreased remarkably by 97, 97, and 98%, respectively (Fig. 4d). These data implied that *CrMAPK3* silencing positively regulated *FOX1*:*ARS* expression. *CrMAPK3* silencing also decreased the gene expression of endogenous *FOX1* in *Chlamydomonas*. The mRNA levels of *CrFOX1* of the RNAi11 strain decreased by 63, 54, and 71% when this strain was cultured for 12, 24, and 48 h under the –Fe condition, respectively. *CrMAPK3* silencing also repressed the iron-induced upregulation of *CrFOX1* gene expression. Therefore, *CrMAPK3* positively regulated the endogenous expression of *CrFOX1* (Fig. 4e).

### CrMAPK3 positively regulates the expression levels of iron uptake-associated genes

**Fig. 5:**
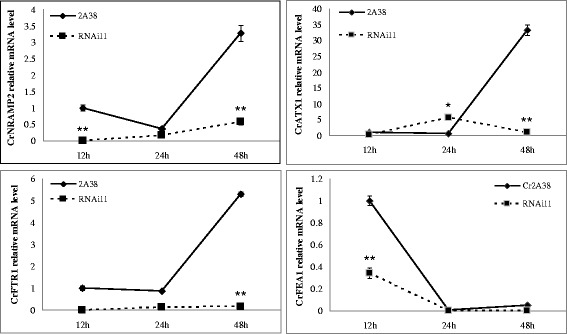
mRNA expressions of iron absorption-related genes in CrMAPK3 RNAi transgenic strain RNAi11 in –Fe. In the CrMAPK3-silenced strain RNAi11, the mRNA levels of genes, including CrNRAMP2, CrATX1, CrFTR1, and CrFEA1, were decreased when the mRNA level of CrMAPK3 was decreased and after they were incubated with –Fe after 48 h. This finding indicated that CrMAPK3 may positively regulate the expression of iron uptake-associated genes, such as CrNRAMP2, CrATX1, CrFTR1, and CrFEA1. The data are shown as means ± SD (*n* = 3). Significance is indicated as *P < 0.05, **P < 0.01

*Chlamydomonas* exhibits an iron uptake pattern similar to Type I plants. During iron deficiency, *Chlamydomonas* cells undergo the affinity iron absorption mechanism by inducing the expression of *CrFOX1* [19], *CrNRAMP2* [18], *CrATX1* [22], *CrFTR1* [20], and *CrFEA1* to enhance iron absorption [18]. *CrFEA1* is located in the cell walls and responsible for the transport of reduced Fe^2+^ via FRE1 (homolog of *Arabidopsis* FRO2) to *CrFOX1*, which is found in the plasma membrane (homolog of yeast FET3 [23]), and reoxidizes Fe^2+^ to Fe^3+^. *CrFOX1* is then transported inside the cells through the plasma membrane protein *CrFTR1*, and ATX1 of yeast transports Cu^2+^ to the cytoplasm. Thus far, direct evidence supporting iron transmission has yet to be obtained, but studies have shown that ATX1 is an iron-deficiency-inducible protein. The *NRAMP* gene family is located on the vacuole membrane, and it shuttles Fe^2+^ between vacuole membranes to maintain the iron concentration in the cytoplasm. The mRNA levels of iron absorption-related genes, such as *CrNRAMP2*, *CrATX1*, *CrFTR1*, and *CrFEA1*, in the *CrMAPK3* RNAi transgenic strain RNAi11 are shown in Fig. 5. The mRNA levels of the genes decreased after the strains were cultivated for 48 h under –Fe. The mRNA level of *CrNRAMP2* was decreased by 86% compared with the control after cultivation for 48 h in –Fe. Similarly, the mRNA levels of *CrATX1*, *CrFTR1*, and *CrFEA1* were decreased by 96, 96, and approximately 53%, respectively. These results indicated the association of *CrMAPK3* of *Chlamydomonas* with the iron metabolism-related genes. In the *CrMAPK3*-silenced strain RNAi11, the mRNA levels of the genes, including *CrNRAMP2*, *CrATX1*, *CrFTR1*, and *CrFEA1*, were also decreased when the mRNA level of *CrMAPK3* was decreased. Thus, *CrMAPK3* might positively regulate the expression of iron-uptake-associated genes, such as *CrNRAMP2*, *CrATX1*, *CrFTR1*, and *CrFEA1*.

## Discussion

MAPKs are widely distributed in eukaryotic organisms, such as yeast, humans, and plants, and are involved in phosphorylation signaling cascades in extracellular amplification and intracellular transduction [23]. The MAPK pathway is responsive to biological and non-biological stress stimuli, hormones, or growth factors and to cell division and apoptosis. Moreover, the MAPK pathway comprises MAPKKK, MAPKK, and MAPK and amplifies signals via subsequent phosphorylation by using protein kinases and by migrating to the nucleus; thus, the extracellular stimuli of membrane receptors are connected to the molecular effectors of the cytoplasm and the nucleus [24, 25]. A few MAPKs, including 20 in *Arabidopsis*, 17 in rice, 19 in corn, 21 in aspen (*Populus*), 17 in tobacco, 16 in tomato, and 26 in apple, have been identified [26–28]. Proteins encoded by MAPKs in different species contain various domains. In *Chlamydomonas*, 3 of TEY, 7 of TDY, 4 of TSY, 1 of TPY, and 1 of TTY exist. In *Arabidopsis*, 8 of TDY and 12 of TEY are present. These diversities of types and kinase domains demonstrate that MAPKs participate in many metabolic activities. Through cluster analysis, we found that TDY and TEY of *Chlamydomonas* kinases are highly homologous to those of *Arabidopsis* kinases possibly because only TDY and TEY domains are found in *Arabidopsis*. Other domains are highly similar to human kinases.

Organisms need iron for respiration, DNA synthesis, and enzyme reactions. Transport systems have been developed for iron absorption because iron balance is vital. Iron regulation, especially iron absorption and transportation, has been extensively investigated, but iron signal response systems have been rarely explored. Iron deficiency in humans causes iron deficiency anemia and adolescent iron deficiency 1 syndrome. Iron is an important element required by the body; excessive or scarce amounts of iron likely cause metabolic disorders; therefore, organisms should have a sophisticated control system to regulate the dynamic balance of iron elements [16].

Iron deficiency is the third-most important limiting factor of plant growth and yield in agriculture. Photosynthetic plants reduce their chlorophyll synthesis and photosynthesis rate under iron-deficient conditions.

In humans, iron deficiency causes anemia. Conversely, excess iron increases the risk of liver disease, heart attack, and hypothyroidism. Iron is also a limiting factor in the growth of some tumor cells, and iron chelators are used clinically to inhibit tumor cell growth. Furthermore, studies on iron MAPK signal cascades have focused on human cancers. Iron deficiency inhibits the mitosis of lung carcinoma cells, melanoma cells, and dysembryoplastic neuroepithelial tumor cells and thus induces cell apoptosis [29–31]. Therefore, iron chelators, desferrioxamine (DFO), and Dp44mT are used to treat these cancers clinically [32, 33]. Iron deficiency signals are also transduced through the activation of JNA and P38 by ASK1 (MAPKKK) to regulate the suspension of the mitotic activity and apoptosis of cancer cells [34].

Plant MAPK gene responses to various stresses have also been detected. In our study, gene expression analysis revealed that 16 MAPK genes in *Chlamydomonas* were involved in response to stress. During iron deficiency, 8 MAPK genes, including *CrMAPK3*, were upregulated. Therefore, *CrMAPK3* possibly responded to iron regulation. These findings were further verified by silencing *CrMAPK3*, and our results demonstrated that the mRNA levels of FOX1-ARS, the enzyme activities of ARS, and the endogenous mRNA level of *CrFOX1* decreased. Therefore, *CrMAPK3* positively regulated *CrFOX1* expression. The mRNA levels of –Fe-inducing genes, including *CrNRAMP2*, *CrATX1*, *CrFTR1*, and *CrFEA1*, and the expression of *CrMAPK3* were reduced. These findings confirmed that *CrMAPK3* positively regulated the expression of iron-absorption genes. However, the exact proteins upstream and downstream of *CrMAPK3* should be identified to reveal the MAPK pathway of iron deficiency response in *Chlamydomonas*.

## Methods

### Algal strains and culture conditions

*C. reinhardtii* CC425 (mt) was purchased from the *Chlamydomonas* Genetics Center at Duke University. *C. reinhardtii* 2A38 is a transgenic strain with an integrated *Fox1 promoter*:*ARS* chimeric gene in *C. reinhardtii* CC425 genome. Under iron-deficient conditions, the *CrFOX1* promoter in 2A38 strain stimulated the *ARS* gene expression and appeared blue when the XSO4 substrate was added. Liquid cultures were grown in the TAP medium at 26 °C with agitation at 220 rpm under 110 μmol⋅m^−2^s^−1^ of continuous light for 3 days and then to the TAP, TAP-Fe, TAP-N, or TAP + 150 mM NaCl media for various time periods (12, 24, and 36 h). Total RNA was extracted to prepare cDNA for gene cloning and real-time PCR assay. All *Chlamydomonas* strains were cultured in the TAP or deficiency medium of TAP with Hunter’s trace element mix.

### Bioinformatics analysis of MAPK gene family of Chlamydomonas

*Chlamydomonas* MAPK homologous genes were retrieved from *Chlamydomonas* database (https://phytozome.jgi.doe.gov/pz/portal.html) by using the BLAST of *Arabidopsis* MAPK. Multiple sequence alignments were generated using ClustalX 2.1 and MEGA6. The following parameters were predicted: molecular weights and isoelectric points of proteins in Expasy (http://web.expasy.org/compute_pi/); protein structures in SMART; and conserved protein motifs in PROSITE (http://prosite.expasy.org/) and MEME (http://meme.nbcr.net/meme/). The structures of *CrMAPK* genes were generated online by using the Gene Structure Display Server (GSDS) (http://gsds.cbi.pku.edu.cn/), and the homologous chromosome segments were detected using a synteny plot in Plaza (http://bioinformatics.psb.ugent.be/plaza/versions/pico-plaza/synteny/index). The *CrMAPK* genes were subjected to BLAST analysis in Plaza, and their duplication patterns were detected using a synteny plot. The subcellular localization of *Chlamydomonas* MAPKs was performed using Euk-mPLoc2.0 (http://www.csbio.sjtu.edu.cn/bioinf/euk-multi-2/).

### Statistical analyses

Data were presented as mean ± S.D. One-way ANOVA followed by Duncan’s post-test was performed to examine significant differences between means. In all cases, comparisons showing *P* < 0.05 were considered significant.

### mRNA abundance detection

Three independent cell populations exposed to different stress conditions in various periods were collected. Total RNA was extracted and real-time PCR was performed as described previously [35]. The primers used in this study are listed in Tables 2 and 3. All the products were 300 +/- 50 nt The amplification rate of each transcript (Ct) was calculated using the PCR Base Line Subtracted method performed in the iCycler software at a constant fluorescence level. Cts were determined with three repeats. Relative fold differences were calculated on the basis of the relative quantification analytical method (2^-ΔΔCT^) using 18 s rRNA amplification as internal standard [36].

**Table 2:** Primer sequences for amplifying the Chlamydomonas MAPK genes

Primer Name	Primer Sequences
CrMAPK2-F	GAGGCAAACCGATACACGAT
CrMAPK2-R	CGAGTACTTGGCGAAGAAGG
CrMAPK3-F	CGTCCGCAAAAGACAGTGTA
CrMAPK3-R	GGAGCACCTGGTAGACGAAG
CrMAPK4-F	GCACAGCCTCATAGGAAAGG
CrMAPK4-R	CCAATCACTGTGTGCAGGTC
CrMAPK5-F	GGAGGTGCACAAGCAGTACA
CrMAPK5-R	GTGCTACCCAGCAGGATCTC
CrMAPK6-F	CCAGCTCAAGCTCATCATCA
CrMAPK6-R	CATCTCGTCAAAGTCCAGCA
CrMAPK7-F	AAGACCGGGACAAGTTCCTT
CrMAPK7-R	ATGTACAGCTCCGCCATGAT
CrMAPK8-F	AGAGATTTGAAGCCCAGCAA
CrMAPK8-R	ACCTTGGTGATGAGGGACAG
CrMAPK9-F	GCTGTGCGACTTTGGCTTTGC
CrMAPK9-R	TACTTGCGGTCCAGCGTCTCCG
CrMAPK10-F	CGTTGTGGCGGTGAAAGG
CrMAPK10-R	GAACCCGAAGTCGCACAGC
CrMAPK11-F	ATCAAGCCCGCCAACATCC
CrMAPK11-R	GTTGTCAGATACCAGCACCTCC
CrMAPK12-F	GATGCCTTCAAATCTAAAACCG
CrMAPK12-R	AAGTCGCACAGCCGCACCAC
CrMAPK13-F	TCGGGCACGCCGCTGTTC
CrMAPK13-R	TAGCGTGCGACCTGGTGCGG
CrMAPK14-F	GGGCGTATGGGTCGGTAT
CrMAPK14-R	GCAGGTTGACGATGTTCAC
CrMAPK15-F	CACGATTGATGCGATAGAGGAG
CrMAPK15-R	AAGGTCGGGAACGGTGGA
CrMAPK16-F	TGGCAGTTAGCCTCCGTCATT
CrMAPK16-R	CGCAAAGCCAAAGTCGCA
CrMAPK17-F	GAGGTTACCGTGCTCAATGGC
CrMAPK17-R	GGGTAGCGGTCCTCCAACA

**Table 3:** Primer sequences for amplifying Chlamydomonas iron responsive genes

Gene Name	Locus Name	Primer Name	Primer Sequences
CrARS2	Cre16.g671350	ARS2-F	ATGGGTGCCCTCGCGGTGTTC
		ARS2-R	GTAGCGGATGTACTTGTGCAG
CrFOX1	Cre09.g393150	FOX1-F	GACGTGGAGGCCCAGAAG
		FOX1-R	CGCGACGAAGTAGGTGTTG
CrFTR1	Cre03.g192050	FTR1-F	TCTTTCGGGAGACCATTGAG
		FTR1-R	GAAGCATAGCAAAGCCAAGG
CrFEA1	Cre12.g546550	FEA1-F	CTCAAGTACCACCTGCACGA
		FEA1-R	ACATAGCTCTTGCCGAGGAA
CrATX1	Cre09.g392467	ATX1-F	AGCTCGTGTCCTCGTAAAGC
		ATX1-R	CTGCAACAGGTTCCGTGTAA
CrNRAMP2	Cre07.g321951	NRAMP2-F	CTGTCGCAGGTGATCCTGT
		NRAMP2-R	TTTGCACCACCAGGTTAATG

### Construction of CrMAPK3 RNA interference vector and transformation of Chlamydomonas

Using *Chlamydomonas* cDNA as a template, we amplified the fragments through PCR with forward primer *CrMAPK3*-F: CGTCCGCAAAAGACAGTGTA and reverse primer *CrMAPK3*-R: CTTCGTCTACCAGGTGCTCC. We then inserted the amplified fragments into pMD18-T vector to generate *CrMAPK3*-18 T, which was further digested with HindIII and BamHI and ligated into the intermediate vector T282 to produce *CrMAPK3*-T282. *CrMAPK3*-T282-*CrMAPK3* with inverted repeat sequence of *CrMAPK3* (*CrMAPK3IR*) was developed by digesting *CrMAPK3*-18 T and *CrMAPK3*-T282. *CrMAPK3IR* was inserted into EcoRI-digested *pMaa7*/*XIR* to produce *Maa7IR*/*CrMAPK3IR. Maa7IR*/*CrMAPK3IR* was then transformed into *C. reinhardtii* 2A38 by applying the glass bead procedure [37].

### ARS (arylsulfatase) activity detection

ARS activity was determined as described by Davies and Grossman [38]. XSO4 (10 mM) was added to plates with –Fe TAP solid medium and scribed before clones were inoculated. After 1 day, the transformants that expressed ARS activity were identified using blue halos around their colonies. The cells were initially collected by centrifugation to quantify the ARS activity. The supernatant was mixed with 0.1 M glycine–NaOH at pH 9.0, 10 mM imidazole, and 4.5 mM *p*-nitrophenyl sulfate. The reaction mixture was incubated at 27 °C for 30 min. The reaction was terminated by adding 0.25 M NaOH, and its absorbance at 410 nm was determined. A standard curve of *p*-nitrophenol (Sigma Chemical Co.) was obtained using 0.2 M NaOH.

## Conclusions

Silencing *CrMAPK3* decreased the mRNA levels and ARS activities of FOX1:ARS chimeric gene and endogenous *CrFOX1*. The mRNA levels of iron-responsive genes, such as *CrNRAMP2*, *CrATX1*, *CrFTR1*, and *CrFEA1*, were also remarkably reduced. Therefore, *CrMAPK3* regulated the expression of iron-deficiency-responsive genes in *C. reinhardtii*.
